# Utilization of D-dimer assay, CT angiography, and the incidence of pulmonary embolism in the hospital district of Helsinki and Uusimaa (2011–2017)

**DOI:** 10.1007/s11239-022-02698-2

**Published:** 2022-09-18

**Authors:** Markus Sane

**Affiliations:** Helsinki University Central Hospital: Helsingin seudun yliopistollinen keskussairaala, Helsinki, Finland

## Background

The diagnosis of PE is commonly established with computed tomography pulmonary angiography (CTPA) and its use along with the PE incidence has increased substantially in the 21st century [1, 2]. Several explanations for the increased PE incidence has been proposed such as higher prevalence of risk factors including cancer, long haul air travel, obesity and diabetes [3]. However, the high ability of CTPA to also detect smaller subsegmental PEs has indicated potential overdiagnosis of PE [1]. Unfortunately, exact data on CTPA use has not been previously reported with the PE incidence.

The aim of this study was to compare PE incidence trend from the hospital district of Helsinki and Uusimaa (HUS district) during 2011–2017 with the exact data on the use of CTPA examinations and D-Dimer tests.

## Methods

The HUS district is the biggest hospital district in Finland organizing specialized health care including laboratory and imaging services for approximately 1.7 million inhabitants. The healthcare system in Finland is universal with excellent coverage and a full electronic patient record system has been used the whole 21st century. Noteworthy in HUS district the PE diagnoses are almost entirely made in public hospitals with uniform electronic patient record system and compilation of hospital discharge data.

The yearly incidence data of PE was collected from the Care Register for Health Care [4]. This database collects social security code- linked information of both inpatient and outpatient use of health services. Up to four different diagnoses for each discharge can be listedand the number of patients with International Classification of Diseases (ICD) code I26.0 or I26.9 among their discharge diagnoses were identified in age quartiles. The data was collected between 2011 and 2017.

The quantity of annual CTPA examination and D-dimer tests (other than point of Care (POC) tests) were available from the HUS Diagnostic Center responsible for imaging and laboratory services for the whole population in HUS district. The different CT examinations and laboratory tests can be identified with unique code and exact quantities on CTPA examinations and D-dimer tests performed and analyzed annually were collected.

The PE incidence and the quantities of CTPA examinations and D-dimer tests are presented in proportions per 100 000 inhabitants and the population in HUS district at the end of each year was used as denominator. The PE incidence is also presented per age quartiles.

The association between PE incidence and quantities of CTPA examinations as well as the quantities of D-dimer tests and CTPA examinations were evaluated with the linear regression.

## Results

In total, 10 559 subjects with PE among their discharge diagnoses were identified during the study period with an average annual PE incidence of 94/100 000 population (Table 1). At the same time 24 276 and 218 163 CTPA examinations and D-Dimer test were registered, respectively. The association between PE incidence and CTPA examinations was not statistically significant (p = 0.9).

The estimated yield of CTPA examinations decreased from 50% (1369/2754) to 31.5% (1400/ 4439) during the follow-up period.

**Table 1:** The number of identified PE cases and number of individuals in age quartiles. Pulmonary embolism incidence in age quartiles in HUS district 2011–2017

Year	Pulmonary embolism Incidence per 100 000 inhabitants				
	Individuals 0–24 years (PE cases/ population)	Individuals 25–49 years (PE cases/ population)	Individuals 50–74 years (PE cases/ population)	Individuals 75- years (PE cases/ population)	Total population
2011	7.0 (32/454,023)	46 (262/563,744)	171 (746/434,933)	446 (411/92,154)	94 (1451/1,544,854)
2012	9.2 (42/457,522)	41 (233/567,427)	169 (748/442,799)	408 (387/94,881)	90 (1410/1,562,629)
2013	7.0 (32/459,940)	47 (270/572,070)	185 (834/450,992)	494 (486/98,291)	103 (1622/1,581,293)
2014	6.7 (31/462,058)	43 (248/576,540)	178 (815/458,784)	464 (473/101,834)	98 (1567/1,599,216)
2015	5.8 (27/463,041)	43 (253/581,728)	180 (839/467,079)	395 (412/104,268)	95 (1531/1,616,116)
2016	4.1 (19/464,340)	48 (282/587,821)	191 (904/472,032)	380 (418/109,910)	99 (1623/1,634,103)
2017	4.1 (19/464,569)	38 (229/594,327)	161 (776/480,446)	295 (331/112,148)	82 (1355/1,651,490)

82

**Fig. 1:**
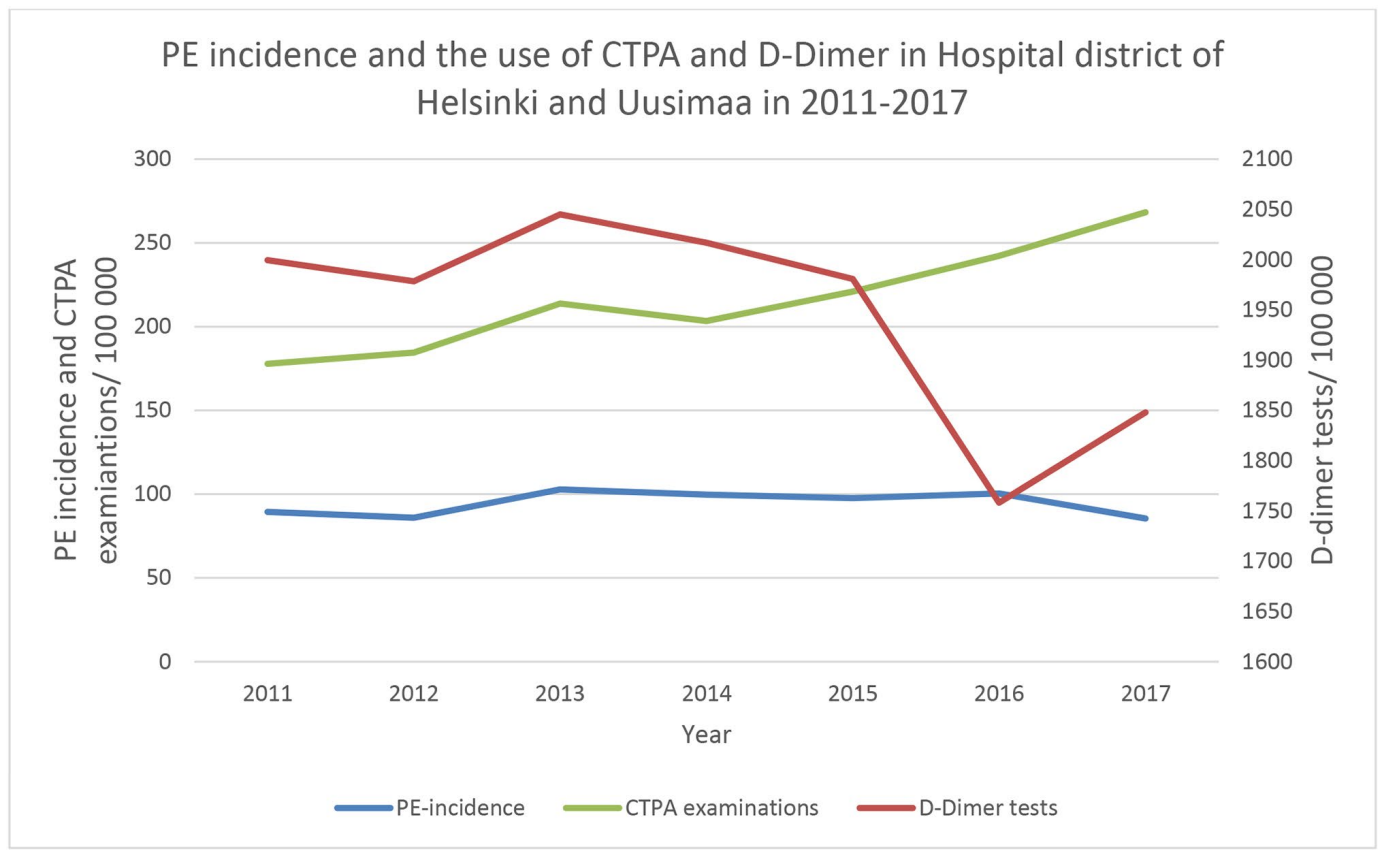
Annual PE incidence and the use of CTPA examinations per 100 000 population and D-dimer tests in HUS district

The number of CTPA examinations per 100 000 inhabitants increased 50.8% from (177.8) in 2011 to (268.2) 2017 whereas the use of D-dimer tests analyzed in laboratory decreased 10% from 1999.5 to 1847.9, respectively. The association was not statistically significant (p = 0.07).

## Discussion

This study showed that in HUS district with a population of approximately 1.7 million the PE incidence has plateaued since 2011 although at the same time the use of CTPA has increased 50%. The average annual PE incidence in HUS district was in line with the recent results from Germany from 2015 [2].

Previously it has been thought that the more CTPA examinations are performed the more clinically insignificant PEs can be found leading to increased PE incidence [1]. This has indicated potential overdiagnosis of PE [5] and aroused debate of its existence [6, 7]. This data provides exact quantities of CTPA use along with the PE incidence and significant association with the PE incidence and CTPA use was not seen (p = 0.9). The increased use of CTPA may be explained by the change of the paradigm in PE diagnosis which has shifted from diagnosing PE from high risk patients to the exclusion of PE in low risk patients [1]. Although this may not lead to overdiagnosis of PE it has several downsides such as predisposing patients to unnecessary radiation and costs to healthcare [1].

The observation that D-Dimer tests analyzed in the laboratory decreased nearly 10% is interesting and may also be associated with the increased use of CTPA examinations. The low specificity of D-dimer test is an acknowledged challenge but several solutions have recently been introduced to enhance the diagnostic yield of CTPA examinations without causing risk for missing PEs [1]. Unfortunately the data has shown that adherence to guidelines has been poor causing as low as 1% diagnostic yields for PE when data from single center has been analyzed [8]. To note, this study was unable to evaluate the proportion of PE diagnosis made with other modalities such as ventilation perfusion scintigraphy or other CT examinations but the estimated yield in HUS district decreased from 50 to 30% during the study period, which is slightly higher than in large multicenter study of 15 Emergency departments from Australasian where CTPA yield for PE was on average 15% [9]. This study was unable to evaluate the adherence to guidelines by investigating the proportion of CTPA examinations without preceding D-dimer test for subjects with low or moderate pretest probability for PE. However the possible association with D-dimer testing and CTPA examinations highlights that the use of these two important means in PE diagnosis should be closely monitored in centers where PE diagnostics are performed.

The limitations of registry based study such as miscoding, under coverage and possibility of duplicate cases should be considered when the results of this study is interpreted. However certain aspects in the Finnish health care system, the registry used and the study aim ensures that the results can be considered reliable.

Firstly, as the study analyzed PE incidence and most common diagnostic tools used in PE diagnosis from a single region with universal health care and uniform electronic patient record systems which were unchanged during the study period the impact of miscoding can be considered low.

In addition the possibility of listing up to four discharge diagnoses for certain hospital admission also decreases the risk of under coverage of PE diagnoses but as the data was social security code linked the chance of duplicate cases can also be avoided. The Care Register for Health Care also has a long history and it is one of the oldest individual level registry covering the whole country in the world [10]. Its performance has been analyzed in recent systematic review which found that more than 95% of the discharges could be identified from the registry and the positive predictive value for common diagnosis was 75–99%,- unfortunately the positive predictive value of PE diagnosis was not evaluated [10].

The PE diagnosis can be generally only made in hospitals equipped with CT scanners. The structure of the healthcare in Finland ensures that almost in all cases the diagnosis has been made in public hospital and as the imaging services in HUS district are provided only by HUS Diagnostic Center the exact data on the quantities of CTPAs performed can be acquired. Finally, the study period was relatively short and as the aim of the study was mainly analyze the relationship of PE incidence and CTPA use it is likely that the possible limitations related to registry studies does influence impact less on this aspect.

Noteworthy, the data of D-dimer use did not include the POC D-dimer tests and possibly these have replaced the use of D-dimer tests analyzed in laboratories. Therefore the speculation on the association with decreasing D-dimer testing to CTPA use may be incorrect. However as the D-dimer is the main laboratory test used in PE exclusion the data was presented in connection with PE incidence and CTPA use.

This study showed that PE incidence plateaued in HUS district with a 1.7 million population during 2011 and 2017. This has occurred despite the CTPA use has increased markedly which is in conflict with the presented PE -overdiagnosis hypotheses. However, it is concerning that the yield of CTPA examinations has decreased 40% during the follow up period which highlights the importance of utilizing the diagnostic algorithms in PE diagnosis [1].
